# Echoes of old HIV paradigms: reassessing the problem of engaging men in HIV testing and treatment through women’s perspectives

**DOI:** 10.1186/s12978-017-0387-1

**Published:** 2017-10-05

**Authors:** Leila Katirayi, Addmore Chadambuka, Auxilia Muchedzi, Allan Ahimbisibwe, Reuben Musarandega, Godfrey Woelk, Thorkild Tylleskar, Karen Marie Moland

**Affiliations:** 1 Elizabeth Glaser Pediatric AIDS Foundation, 1140 Ave NW, Suite 200, Washington, D.C, CT 20036 USA; 2 Elizabeth Glaser Pediatric AIDS Foundation, Harare, Zimbabwe; 3 Elizabeth Glaser Pediatric AIDS Foundation, Lilongwe, Malawi; 4 Center for International Health/CISMAC (Centre for Intervention Science in Maternal and Child Health), University of Bergen, Bergen, Norway

**Keywords:** HIV, PMTCT, ART initiation, Qualitative, Universal treatment, Africa, Lifelong treatment, Male engagement

## Abstract

**Background:**

With the introduction of 2016 World Health Organization guidelines recommending universal antiretroviral therapy (ART), there has been increased recognition of the lack of men engaging in HIV testing and treatment. Studies in sub-Saharan Africa indicate there have been challenges engaging men in HIV testing and HIV-positive men into treatment.

**Methods:**

This qualitative study explored women’s perspective of their male partner’s attitudes towards HIV and ART and how it shapes woman’s experience with ART. Data were collected through in-depth interviews and focus group discussions with HIV-positive pregnant and postpartum women on Option B+ and health care workers in Malawi and Zimbabwe. In Malawi, 19 in-depth interviews and 12 focus group discussions were conducted from September–December 2013. In Zimbabwe, 15 in-depth interviews and 21 focus-group discussions were conducted from July 2014–March 2014.

**Results:**

The findings highlighted that many men discourage their partners from initiating or adhering to ART. One of the main findings indicated that despite the many advancements in HIV care and ART regimens, there are still many lingering negative beliefs about HIV and ART from the earlier days of the epidemic. In addition to existing theories explaining men’s resistance to/absence in HIV testing and treatment as a threat to their masculinity or because of female-focused health facilities, this paper argues that men’s aversion to HIV may be a result of old beliefs about HIV and ART which have not been addressed.

**Conclusions:**

Due to lack of accurate and up to date information about HIV and ART, many men discourage their female partners from initiating and adhering to ART. The effect of lingering and outdated beliefs about HIV and ART needs to be addressed through strengthened communication about developments in HIV care and treatment. Universal ART offers a unique opportunity to curb the epidemic, but successful implementation of these new guidelines is dependent on ART initiation and adherence by both women and men. Strengthening men’s understanding about HIV and ART will greatly enhance women’s ability to initiate and adhere to ART and improve men’s health.

## Plain English summary

Many people face challenges starting and remaining on antiretroviral treatment (ART), the medication recommended for those with human immunodeficiency virus (HIV). In sub-Saharan Africa, men greatly influence their partner’s decisions, including decisions regarding health care. This paper explores women’s perspective of their male partner’s attitudes towards HIV and ART and how it shapes woman’s experience with ART. Data were collected using individual in-depth interviews and focus group discussions of 6–12 people. Data were collected with HIV-positive pregnant and postpartum women and health care workers in two countries in sub-Saharan Africa, Malawi and Zimbabwe. The findings highlighted that across the study settings women commonly experience their male partners discouraging them from initiating and adhering to ART. One of the main findings indicated that despite the many advancements in HIV care and ART regimens, there are still many lingering negative beliefs about HIV and ART from the earlier days of the epidemic. This study argues that lack of accurate information about HIV and ART may contribute to male partners failing to engage in HIV testing and treatment and supporting their female partner’s initiation and adherence to ART. Hence, strengthening men’s understanding about HIV and ART may greatly enhance women’s ability to initiate and adhere to ART and at the same time improve men’s health.

## Background

### HIV treatment in sub-Saharan Africa

Sub-Saharan Africa has witnessed an expansion in the coverage of HIV antiretroviral treatment (ART) to record numbers of people, increasing from 4.1 million in 2010 to 10.3 million 2015. Treatment coverage increased from 24% in 2010 to 54% in 2015 [1]. A challenge to increasing ART initiation is that only 45% [39–62%] of people living with HIV in sub-Saharan Africa know their HIV status. Within sub-Saharan Africa, 67% [65–68%] of men and 57% [55–60%] of women living with HIV are not receiving antiretroviral therapy [2].

### Universal ART

Under the 2013 World Health Organization (WHO) treatment guidelines, only those with a CD4 count less than 500 cells/mm^3^ or WHO clinical stage 3/4 were recommended to start ART [3]. However, the 2013 guidelines for prevention of mother-to-child HIV transmission (PMTCT) recommended that all HIV-positive pregnant and breastfeeding women initiate ART regardless of CD4 cell count or clinical stage for at least the duration of mother-to-child transmission (MTCT) risk, and in high HIV burden settings, initiate life-long ART (referred to as “Option B+”). In September 2015, the WHO released new recommendations, referred to as “treatment for all” or “universal ART,” stating that ART should be initiated in everyone living with HIV regardless of CD4 cell count or clinical stage [4]. In 2015, an estimated 36.7 million people were living with HIV, with 17 million receiving ART [1]. With the new guidelines, there is expected to be a significant increase in the number of people who will now be eligible for ART.

Universal ART falls under a concept labelled “Treatment as Prevention” (TasP) which has become a driving rationale for HIV policy [5]. Modelling studies have suggested that expansion of ART coverage could be effective in reducing transmission at the population level [6]. The 2015 WHO guidelines could change the face of the HIV epidemic and significantly decrease HIV incidence, depending on the success of ART uptake and retention.

Previous research has demonstrated challenges with initiating seemingly healthy (asymptomatic) individuals on lifelong ART irrespective of their CD4 cell count. A study in Canada found that 30.5% of HIV-positive patients opted to defer ART, with the most common reason for delaying ART among those with CD4 ≥ 500/mm^3^ cited as “no urgency” or “no need for treatment” [7]. A study in Uganda found that those who had been HIV-positive for some time and had become eligible for ART under the new “universal ART” guidelines were more likely to delay their decision to start ART compared to those who were newly diagnosed. The change in guidelines to universal ART conflicted with the information they had previously received regarding when to start ART. These participants were proud of their ability to maintain a high CD4 count and regarded delaying ART initiation as a major accomplishment [8]. At the time of writing, some countries have begun implementing universal ART as national policy; however, little is known about the effects of universal ART on the social dynamics of disclosure processes, adherence and retention [9].

### Challenges engaging men in HIV testing and treatment

Universal ART guidelines will be the first effort to initiate men on ART with CD4 ≥ 500/mm^3^. Previous studies have demonstrated the challenges of getting men to access health facilities, test for HIV and initiate and adhere to ART [10–12]. Masculinity norms discourage men from seeking care in an effort to ‘tough it out’ [13, 14]. Another challenge is that men view the health facility as ‘a woman’s domain’ making it uncomfortable for men to access services [14].

Men are generally initiating ART much later and have worse outcomes than women [15]. One study from South Africa found that men had 4.4-higher odds of being untested, and HIV-positive men had 1.8 and 1.6-higher odds of being unaware of HIV status and untreated, respectively, compared to women [16]. Previous studies have reported that men only access HIV services when in late stage disease [17–19]. More men in sub-Saharan Africa die from acquired immunodeficiency syndrome (AIDS) than women, despite women having higher HIV prevalence than men [10]. As stated in a paper by Dovel, “AIDS prevalence may have the face of a woman, but AIDS mortality has the face of a man” ([10], page 1).

With the history of male reluctance to undergo HIV testing and receive treatment, there is an urgent need to tailor programs to the needs of men. Hence, focusing on the role of men, this paper aims to identify the lessons learned from implementation of lifelong ART under Option B+ to inform the scaling up of universal ART. HIV-positive women’s experiences with their male partners is used an entry point to understand the challenges of HIV testing and treatment and how men affect their partner’s ability to initiate and adhere to ART.

## Methods

### Study design

This descriptive qualitative study in Malawi and Zimbabwe collected data using in-depth interviews (IDIs) and focus group discussions (FGDs) to explore the acceptability of initiating lifelong ART with pregnant and breastfeeding women. As the first country to pilot Option B+, Malawi was selected for the study. Zimbabwe was selected as a comparative case for its interest in assessing the acceptability of Option B+ at pilot sites. The study was initially conducted in Malawi in 2013 and then modified for Zimbabwe’s context and conducted in Zimbabwe in 2014. Results from Malawi have been published [20]. The primary focus of the studies in Malawi and Zimbabwe was the acceptability of Option B+. During analysis it was discovered that both data sets had in-depth information about women’s perspectives about their male partners’ attitudes towards HIV and ART. This data provided a unique opportunity to explore how women understand and interpret men’s attitudes towards HIV and ART and how it shapes the woman’s experience with ART. Results illustrating the challenges and fears experienced by men as perceived by the pregnant and postpartum partners and the influence of the male partner on women’s ability to initiate and adhere to ART are presented here. While the study was conducted in different contexts, findings were very similar and therefore have been combined in this paper to strengthen the transferability of the results. Comparing the results of the two country contexts drew attention to the challenges men are facing to engage in HIV testing and treatment. The findings would be most applicable in a low-income setting, with a patriarchal society and over-stressed health care systems.

### Study population

In both Malawi and Zimbabwe, the study population was comprised of pregnant and postpartum women initiated on Option B+ and health care workers (HCWs) who provided PMTCT services. Pregnant women were at least 18 years of age, HIV-positive, receiving the ART regimen associated with Option B+ and receiving antenatal care (ANC) from one of the selected study sites. Postpartum women met the same selection criteria in addition to having delivered and breastfed a child within the last 18 months. HCWs had to have worked in the selected health care facilities for the previous six months providing maternal and child health services, including PMTCT.

### Site selection

In Malawi, four health care facilities were selected as the study sites. The districts of Lilongwe, Dedza and Mchinji were purposively selected for their rural, urban and peri-urban settings. In addition, different facility types including government-owned (free services) or privately-owned (pay-for services) were selected. Within these parameters, the sites with the highest volume of HIV-positive pregnant women were selected.

In Zimbabwe, the urban and rural districts of Harare and Zvimba were selected based on their relatively close proximity to the city of Harare. Once the districts were chosen, eight public health facilities (free services) with the highest annual volume of HIV-positive pregnant women were selected.

### Malawi & Zimbabwe setting

Malawi and Zimbabwe share similar characteristics of predominantly patriarchal societies where the male partner is considered the ‘head of the house’ and women’s ability to make decisions for themselves or family is limited [21, 22]. In a study in Malawi and Zimbabwe participants reported the wife is supposed to be seen as ‘silent, subordinate and submissive.’ “Culture says that she has to be submissive because the head of the house is a man, he is the breadwinner, he takes care of the family. No decision can be made without consulting the man, so she is in a submissive kind of situation.” (p.182) [23]. Male dominant attitudes limit a woman’s choices about her sexuality, use of contraception, decisions around having children, the welfare of her and the children and control over household finances and resources [24].

Malawi and Zimbabwe have similar population sizes, Malawi has a population of approximately 18,300,000 and Zimbabwe a population of approximately 16,300,000 [25]. Malawi has nine main ethnic groups while Zimbabwe is more homogenous with two primary ethnic groups. Both Malawi and Zimbabwe are predominantly Christian. Malawi has a higher fertility rate of 4.9 children per woman, compared to 3.7 children per woman in Zimbabwe [26].

Malawi has an adult (15–49 years) HIV prevalence of 10.0% [27]. Zimbabwe has an adult HIV prevalence of 14.7% [28]. In Zimbabwe, the HIV prevalence among pregnant women 15–49 years has declined from 16.1% in 2009 to 15.9% in 2012 [29]. In 2010 in Malawi, the HIV prevalence among HIV-positive pregnant women was reported to be 10.6% [30]. In both countries eight of ten HIV-positive pregnant women access ART [28, 31]. In 2015 Malawi saw an estimated 4800 new infections among children, while Zimbabwe had an estimated 4900. In 2015, approximately 15,000 women acquired HIV in Malawi compared 32,000 women in Zimbabwe. According to the UNAIDS 2016 estimates, the overall MTCT rate is reported to be 9% in Malawi [31] and 7% in Zimbabwe [28].

### Data collection

Data were collected in Malawi during September –December 2013 and in Zimbabwe from July 2014–March 2015 with trained research assistants (RAs). Reflexivity was discussed during the data collection training and RAs were trained to reflect upon their pre-understanding of the study topic areas and how that could influence how they asked questions and selected follow-up questions, potentially introducing bias. Pregnant and postpartum women were selected using convenience sampling, they were﻿ recruited as they arrived at the health facility. HCWs identified eligible women and referred them to onsite RAs.

Pregnant and post-partum women participated in both IDIs and FGDs, while HCWs only participated in FGDs. Eligible HCWs were identified by the nurse in-charge and referred to the RAs. All eligible HCWs were invited to participate in FGDs in both Malawi and Zimbabwe. All study participants voluntarily agreed and signed written informed consent forms. Confidentiality was discussed during the informed consent process and at the beginning of the FGDs.

The data collection tools used in Malawi were adapted to fit the Zimbabwe context. Both versions of the data collection tools for the pregnant and postpartum women focused on the following: understanding general perceptions towards lifelong ART; messages provided at the clinic by HCWs; perspectives in the community; barriers and facilitators to initiating and adhering to ART; male involvement; disclosure, and support received. The FGD guides for the HCW asked about aforementioned items in addition to asking about the HCW’s preparation and attitudes towards providing Option B+. Single interviewers conducted the IDIs; a moderator with a note-taker conducted the FGDs which consisted of 6–12 participants. IDIs with pregnant and postpartum women took approximately 65 minutes , FGDs with pregnant and postpartum women averaged two hours and FGDs with HCWs averaged two hours and 35 minutes. In Malawi data were collected in the local language of Chichewa, and Zimbabwe data was collected in the local language of Shona (although some FGDs with HCWs were conducted in English). All IDIs and FGDs were audio-recorded to ensure that the data were accurately captured without subjective filtering of information. Audio-recordings were simultaneously transcribed and translated, including descriptions of the participant’s body language and non-verbal response. The study coordinator reviewed approximately 10% of the transcripts while listening to the audio-recording to ensure the translations were accurate.

### Data analysis

Data from the studies conducted in Malawi and Zimbabwe were analyzed separately using thematic analysis. All transcripts were entered into MAXqda v.10. Transcripts were reviewed by the study team, and a code list representative of the findings was created. The code lists were circulated among each study team, including the in-country staff to ensure that it accurately reflected the data. The code list was updated several times based on feedback. Related codes were grouped into overarching themes. Data were analyzed by study population. Data reduction and summary tables were created to organize the results and track the emergence of themes from the data. Results from both countries that focused on women's perspectives of men’s attitude towards HIV testing and treatment and how men influenced their partner’s ART initiation and adherence were reviewed using the data reduction and summary tables and code reports. Quotes were selected from both countries to illustrate thematic findings.

### Ethical considerations

Ethical approval was received in Malawi from the Malawi Ministry of Health National Health Sciences Research Committee. In Zimbabwe, the study received ethical approval from the Medical Research Council of Zimbabwe.

## Results

### Study population

In Malawi, a total of 19 IDIs were conducted with pregnant women and 20 with postpartum women. Four FGDs were conducted with HCWs, four FGDs with pregnant women and four FGDs with postpartum women. In Zimbabwe 15 IDIs were conducted with pregnant women and 19 IDIs were conducted with postpartum women. Four FGDs were conducted with HCWs, eight FGDs with pregnant women and nine FGDs with postpartum women.

In both countries, the majority of participants were married. The mean age for women in Malawi was 28.3 and 28.8 years in Zimbabwe. In Malawi, most participating women had completed primary education, while in Zimbabwe most participating women had completed secondary education (Table 1). In Malawi and Zimbabwe, most HCWs had 1–10 years of experience. In Malawi, the majority of HCWs were nurses, while in Zimbabwe only nurses participated in the study (Table 2).

**Table 1:** Demographic characteristics for women that participated in IDIs and FGDs in Malawi & Zimbabwe

	Malawi		Zimbabwe	
	Pregnant Woman *N* = 38 n (%)	Postpartum Woman *N* = 94 n (%)	Pregnant Women *N* = 70 n (%)	Postpartum Women *N* = 96 n (%)
Age (years)				
< 25	9 (24%)	23 (24%)	10 (14%)	25 (26%)
25–34	22 (58%)	59 (63%)	54 (77%)	57 (59%)
35–44	7 (18%)	12 (13%)	6 (9%)	14 (15%)
Married/Cohabitating	34 (89%)	74 (89%)	65 (93%)	86 (90%)
Single/Divorced/Widow	4 (11%)	20 (11%)	5 (7%)	10 (10%)
No education	5 (13%)	15 (16%)	4 (6%)	2 (2%)
Primary	22 (58%)	51 (54%)	19 (27%)	26 (27%)
Secondary	11 (29%)	27 (29%)^a^	47 (67%)	68 (71%)

^a^For Malawi postpartum, one woman was missing education level

**Table 2:** Demographic characteristics for the participating health care workers

	Health Care Workers in Malawi *N* = 43 n (%)	Health Care Workers In Zimbabwe *N* = 25 n (%)
Age (in years)		
< 25	0	1 (4)
25–34	19 (44)	6 (24)
35–44	15 (35)	14 (56)
45+	9 (21)	4 (16)
Level of qualification (Malawi)		
ART Clerk	3 (7)	–
Nurse	27 (63)	–
Clinical Officer	6 (14)	–
HSA^a^	4 (9)	–
Medical Assistant	2 (5)	–
Counselor (Zimbabwe)	1 (2)	–
Primary Nurse	–	15 (60)
General Nurse	–	9 (36)
Community Nurse	–	1 (4)
Years in current position		
1–5	11 (26)	9 (36)
6–10	13 (30)	10 (40)
11+	19 (44)	6 (24)

^a^Health Surveillance Assistant

### Findings

The results have been organized into five main themes: men’s reluctance to know and share their HIV diagnosis; pretending to be HIV-negative; lacking male spaces; men’s powerful influence on their partner’s ART adherence; and lingering beliefs about HIV and ART (Table 3).

**Table 3:** Summary of findings

Men’s reluctance to know and share their HIV diagnosis • Women felt that men need more information about the benefits of HIV testing • Men refused to receive HIV tests even when feeling unwell or partner was HIV-positive • Men want to “test by proxy” and determine their HIV status based on their partner’s status
Pretending to be HIV negative • If men do learn their HIV status, they often keep it a secret • A few women reported that their partners hid their HIV status and secretly took ART • The majority of women reported that even if their partner learned their HIV status they would still refuse ART
Lacking male spaces • Participants claimed that men do not access the health facility because they view it as a ‘female space’ and are not comfortable accessing services there • Participants recommended creating a separate space for men at the facility • Some participants recommend having more male HCWs available to speak with the men
Men’s powerful influence on their partner’s ART adherence • Men often discouraged or prevented their partners from initiating or adhering to ART • Some men provided incorrect information about ART to their female partners • Some women reported that their partners were supportive and helped remind them to take ART
Lingering beliefs about HIV and ART • There were many lingering beliefs in the community about HIV being a death sentence and a lack of messages about the ability to live a long and healthy life on ART • Previously ART was for those who had low CD4 counts and were very ill. The idea that ART is only for the very ill remains in the community • Many people are not aware of the developments in ART and believe that ART is still a complicated multi-pill regimen • Many fear the side effect of being disfigured, that was associated with drugs used in the older ART regimens

### Men’s reluctance to know and share their HIV diagnosis

In both Zimbabwe and Malawi, men’s lack of knowledge about HIV was repeatedly mentioned as linked to fear of HIV testing. Some women attributed men’s lack of knowledge to men not utilizing the health facility, as it is mostly women who attend the facility for antenatal care. Women felt that the lack of knowledge was a significant challenge and mentioned the need for the men to be educated on HIV and ART.

“The messages that are given, to me it’s not enough, because it targets the woman who is pregnant, I wish men who are responsible for the pregnancies could also be taught, because if they are not taught, they won’t be concerned at all, especially when it comes to sex, or even reminding their partners to take the medication. They don’t care because they don’t know anything about that, and the day they are angered, they will harass you because of the medication, because they don’t have knowledge.” (Zimbabwe, lactating woman)

“Husbands should have their part to receive counseling because marriages are ending because a woman is receiving medicine and the husband is not accepting because it is difficult for a man to accept a thing from a woman.” (Malawi, lactating)

It was commonly reported in both Zimbabwe and Malawi that men refused to get tested for HIV even when feeling ill. Men avoided getting tested for HIV even when visiting the health facility.

“ …even at the time I told him that he must go and get tested he was very difficult even when he is sick and you tell him to go to the hospital he refuses” (Malawi, lactating woman)

“The day that I got tested for HIV I had given birth already and the nurse said when he comes to bring you food, tell him that the nurses want to talk to him. I told him and he sneaked away from the ward, running away from seeing the nurse. He drove his car and went away. So, from that day he does not want to hear about it.” (Zimbabwe, lactating woman).

Women said that some men had stated that there was no need for them to be tested for HIV; they would determine their HIV status based on their partner’s status. The belief that one’s HIV status can be determined by another’s reflects a lack of knowledge and a determination to avoid testing oneself for HIV.

“Some men just say when the woman gets tested whatever status they are told; for instance, if she is found to be HIV positive, then he says I am also HIV positive and if found to be HIV negative, then he says he is also negative. This is what he says and there is no way to argue with him.” (Zimbabwe, lactating woman)

### Pretending to be ‘HIV-negative’

While many women reported that their male partner refused to engage in HIV testing, women in both countries reported believing that their male partners knew their HIV status but chose to keep it a secret until the woman learned her HIV status. Study participants reported that their partners knew they were HIV-positive but failed to disclose their results.

“In my case, it appears my husband got tested for HIV first and he got to know his status but he did not disclose to me. So, when I got pregnant, got tested for HIV and disclosed my status to him, he responded well and said to me it’s common [to be HIV-positive] and told me to initiate ART but he does not want to be on ART.” (Zimbabwe, Lactating woman)

“You know, this is my first marriage but my husband has been married before. I suppose that is where he got HIV infected. I only discovered much later that he was positive because of my visit to the clinic. Otherwise I would have died of HIV without ever knowing I was HIV positive. These men can be so heartless and deceiving.” (Malawi, pregnant woman)

Some women believed that their male partners had tested positive for HIV and had been secretly taking antiretroviral drugs (ARVs).

“Men are taking their medication secretly, without their wives knowing because the man pretends to be negative until he convinces his wife to get tested, knowing that he is infected. So men are the most cunning ones.” (Zimbabwe, lactating woman)

“I saw that there was medicine in the house but I didn’t know they were ARVs because I don’t know how to read. I just thought he’s getting medicine from the hospital for some problem. He didn’t tell me he was HIV-positive. I suppose he knew I would find out eventually on my own at the hospital. The time I was beginning the process of getting my ARVs, he had already been taking them for a whole year.” (Malawi, pregnant)

While there were several cases of women reporting that their partner was secretly taking ART, it was more commonly reported that men refuse to initiate ART after learning that they are HIV-positive.

“Haa, my husband was not accepting that we were HIV positive, he was denying it, and he did not take medication until his death.” (Zimbabwe, lactating woman)

### Lack of male spaces

Participants spoke frequently about the need to educate men about HIV and ART. Some participants stated that there was the need for more “male friendly” services to engage and educate men.

“ …the main problem which they [men] said was that our hospital is not male friendly, when they have come we don’t have a place to leave them… when they have come to the hospital we put them on the line singing with women which we cannot do” (Malawi, HCW).

A Zimbabwean participant suggested creating a special space for men where they would be comfortable receiving services.

“Create a men’s corner where men can gather and have conversations while waiting for their women. This would also be a place where the men can be given instruction or education on HIV. Men need to be instructed on why and how to take the medication and to remind their partners.” (Zimbabwe, pregnant or postpartum).

Some participants recommended more male HCWs are needed to work with the men.

“More male health workers are needed to talk to the men. Culturally, for women to talk to men it is misconstrued to be women challenging men. If a man speaks to fellow men they can listen.” (Zimbabwe, postpartum)

### Men’s powerful influence on their partner’s ART adherence

References were made in both Malawi and Zimbabwe about the man being the head of the house and having the final say in all decisions regarding his partner and family.

“I think women have a burden because of the setup and culture of Malawi that a woman cannot do a thing on her own when her husband did not accept it. She is not an independent…” (Malawi, HCW).

“I think it has more to do with cultural issues and the roles that we play at home as men. According to our culture the men is the head of the household therefore he has the power to make all the decisions, so he does what he wants” (Zimbabwe, HCW)

Because men have the power in their household to determine women’s health care, their lack of knowledge about HIV and ART greatly affects their partner’s ability to initiate and adhere to ART. Women often demonstrate a commitment to initiate and adhere to ART, but men may discourage or prevent their partners from initiating and adhering to ART.

“I met a certain woman, she tested HIV-positive… and attended antenatal very well, but didn’t take the medicine, she tried explaining to her husband who is a Reverend [religious position] but he told her not to take the medicine they will just pray for her… the woman was more willing to start but failed because of the husband. We tried to give her options like the medicine but she said her husband searches everywhere… he checks her bags to see what she brought from the hospital” (Malawi, HCWs)

Religion has been documented as both a barrier and facilitator to ART adherence. Some religious leaders claim that prayer can cure HIV and that drugs should not be taken. This quote highlights religion as a barrier and the fact that a woman’s partner has the power to determine whether she can take her medication.

Some women reported receiving incorrect advice from their partners encouraging them to stop their medication. Given men’s lack of information about ART, men may be confused and advise their partner incorrectly.

“… When we went home after receiving counseling he said that, ‘you have the liberty to stop taking the medicine once you have given birth.’ But when I called my mother because my mother is a nurse she said that’s not true…” (Malawi, pregnant woman)

Partners were also viewed as a facilitator to ART initiation and adherence. While, women frequently discussed their partner as a barrier to ART initiation and adherence, some women reported that their partner was supportive of them being on ART, provided emotional support and helped to remind them to take their medication.

“…my husband is supportive, he encourages me to take lifelong ARVs and always asks me about the appointment date to receive the drugs and make sure he follows that day and reminds me to come and collect the drugs” (Malawi, lactating woman)

Some participants reported receiving support from their HIV discordant partner.

“We were both tested for HIV and he tested negative and I tested positive. He was not disturbed much at that time because the counsellors sat down with us and taught us. When we got home, it was me who got stressed and I even lost weight. He was even encouraging me to take my medicine and warning me against defaulting, saying if I default, I will die. He would wake up, cook for me and giving me my medicine to take. Even after I gave birth, he knows what time to give medicine to our baby. Before he goes to work, he makes sure he gives our baby medicine.” (Zimbabwe, lactating woman)

Partner’s support was one of the main factors determining ART initiation and adherence. Many of the issues women discussed with their own initiation and adherence were related to their partner’s support or refusal of them taking ART. HCWs in both countries reported that engaging the men was crucial to improving women’s ART initiation and adherence.

“… we should involve men because if we are involving the whole family it means the woman has support so that the woman should initiate ART without any challenges.” (HCW)

### Lingering beliefs about HIV and ART

Throughout the data, it was found that beliefs about HIV and ART from earlier days in the HIV epidemic have not changed despite advancements in HIV care and treatment. Study participants described beliefs they had heard in the community such as HIV is a death sentence, ART is only for very sick people, and ART is a complicated multi-drug regimen. The lack of knowledge about the developments in HIV care and treatment highlight a clear gap in how communities are being informed and educated about changing public health practices.

Many women discussed old sentiments about HIV being a death sentence in the community. These kinds of beliefs were prevalent in both countries and deterred both women and men from initiating and adhering to treatment.

“…the husband was refusing to take the medicine and asked why he should bother taking the medicine when this disease has come for the people [implying death].” (Malawi, HCW)

It was also clear that the association between HIV and death was strong:

“I was afraid because I listened to what people were saying”, that “you die, you die”, but when I took the medication I realized that it was not true and I accepted it [ART] well (Zimbabwe, lactating woman)

In both Zimbabwe and Malawi there were many preconceived notions that ART was only for the very ill and therefore those who “felt healthy” reported challenges initiating ART.

“For some, they take the medicine and just leave them without drinking them because they feel good in their bodies [implying feeling healthy]. But for those who see that their bodies are weak and they are found positive, that’s the time they take the medicine.” (Malawi, pregnant woman)

For most men, if they don’t feel any pain, they will tell you that they are not HIV positive. For instance, my husband says it’s me who is ill and not him. So, he says to me “take your ARVs, I will only take ARVs when I am bed ridden.” But this is a problem to me because my wish is for both of us to be on ART but he insists that he is not ill. (Zimbabwe, lactating woman)

Ideas of ART being for the very ill have also continued to linger through images used at the health facility. Some women commented on the visual depictions at the facility indicating that ART is for the very ill.

“…we entered in a room and HCWs started showing us pictures; the first picture was for a person who was very sick and malnourished before she started ARVs but when she went for HIV testing and started taking the medicine she was improving…” (Malawi, lactating woman)

One of the most challenging aspects of earlier HIV care was the large cocktail of drugs required multiple times a day. The shift of ART regimens under Option B+ from an ART regimen requiring multiple daily pills to a regimen with one daily pill has been encouraging for many.

“My husband has colleagues that he works with who initiated ART some time ago and they were taking many tablets at a time. My husband used to comment saying if he was to test HIV positive, he would be happy to hear that he can wait a little while before initiating ART because of the burden of taking many tablets at a time. Now, he accepts it more because people on ART are now taking just one tablet a day.” (Zimbabwe, lactating woman)

Those who are utilizing the facility, such as pregnant and postpartum women, have the opportunity to learn about advancements in HIV care and treatment. Those who don’t access the facility or have other means of becoming informed (such as having a partner in antenatal care) may not be aware of these new developments.

In both Malawi and Zimbabwe concerns of side-effects from the drugs resulting in disfiguration were mentioned. Stavudine was a drug used in earlier ART regimens which caused disfiguration; however, it has not been communicated widely to the public that this drug is no longer in use.

“It was just hearsay from people who started early days that when a person is taking the ARVs she becomes so bumpy in her head, not attractive face, muscles like weight lifters and many things, pimple on the face, ladies hips goes away but when I started there was no such signs which I saw.” (Malawi, lactating woman)

## Discussion

The results highlight many challenges with men accepting HIV testing and treatment and the powerful influence that men have on their partner’s ability to initiate and adhere to ART. While WHO guidelines recommend that all people diagnosed as HIV-positive initiate ART, there are many challenges that need to be overcome first.

Reduction of HIV transmission on a population level requires successful acceptance of ART by the communities to which these new guidelines are targeted. Fully understanding men’s challenges with HIV testing and treatment is critical to optimize universal ART and ensure initiation and adherence of ART.

Two primary theories have emerged to explain why men do not engage in HIV testing and treatment. The first theory suggests that HIV testing and treatment threatens traditional norms of masculinity [19, 32]. The second theory hypothesizes that health services have been designed for women, and men have been systematically left out [10]. These theories are also influenced by contextual barriers including poverty, stigma and distrustful and difficult relationships with HCWs [33]. The findings of this study build on these two theories and provide an additional perspective; that outdated beliefs about HIV care and treatment deter men from engaging in HIV testing and treatment. Outdated beliefs such as HIV is a death sentence, ART is only for the very ill, ART is a complicated, multi-drug regimen with visible side-effects and the lack of knowledge that one can live a long healthy life on ART discourage men from initiating HIV testing and treatment and supporting their partners.

### Fear of loss of masculinity

Connell defines ‘hegemonic masculinity’ as a social norm both created and reinforced by society [34]. Hegemonic masculinity has been associated with men having multiple sexual partners, being viewed as powerful, risk takers, providers and tough in the face of illness [35]. Study participants reported that men seemed fearful of HIV and ART and would refuse to be tested. The fear of receiving a HIV test is well documented in the literature, with previous studies highlighting fears of loss of masculinity, power, and pride with an HIV-positive test result [11, 17]. Participants reported that even when their male partner was very ill they would still refuse to attend the facility to receive a HIV test or start treatment. Local notions of masculinity disfavor seeking help because it is thought to be a sign of weakness [36, 37]. As a result, men often wait to access the health facility until they are very ill [37].

According to the women and HCWs interviewed, men want to avoid being tested for HIV and potentially receiving a HIV-positive diagnosis. HIV is associated with being weak, vulnerable, and requiring help [17]. This association discourages men from getting tested for HIV [38]. Data from both Malawi and Zimbabwe found that men claimed a HIV test was not necessary since they could infer their HIV status from their partner. Diagnosis by “proxy” is well documented in the literature [19, 37]. On the other hand, some men may learn their HIV status and keep it a secret to avoid threatening their masculinity [17]. This study found that if men do learn their HIV status they may attempt to keep it secret and they may also start treatment secretly to avoid admitting illness and therefore weakness. By dismissing their health needs and taking physical risks, men are legitimizing themselves as the “stronger” sex [39].

Study participants reported that their male partners who either tested HIV-positive or strongly suspected a HIV-positive diagnosis refused to initiate ART. Receiving treatment and being dependent on others can threaten a man’s masculinity [32]. The women in this study reported that not only did men not want to initiate ART themselves, they also discouraged or prevented their partner from taking ART. Previous literature has discussed how men may seek to control their partner and their partner’s actions (such as taking medication) as a means to assert power and therefore reinforce an image of masculinity [32]. HCWs providing female partners with health information (which men may not have access to) creates an element of being excluded and therefore feeling out of control. Study participants reported that their partners were often uneducated about HIV and ART, and participants wished the health care messages would target men or include men in the counseling.

### Female centric health care system

The second main theory explaining men’s aversion to HIV testing and treatment postulates that the health facility has been designed to care for women, and men’s health care needs have been neglected. This theory hypothesizes that men’s poor health and poor health seeking behaviors are a result of the female-centric health care system that has left men out [10].

Study participants reported that men do not feel comfortable at the facility and feel that it is a woman’s space. Clinics are seen to be “female spaces” [17, 37]. A study in South Africa found that men felt that that they did not belong at the health facility, and the study emphasized the need for men to learn about HIV in a “safe space” in which men could talk with one another and listen to experiences of other men [40]. Study participants also discussed the need for a male friendly space.

One study in South Africa found that a man who is seen seeking care at a health facility could lead to gossip about HIV status, since men do not have the same reasons as women (i.e. pregnancy) for being seen when healthy [36]. Such perceptions about the health facility may deter men from accessing the facility, which is where the majority of the population receives HIV tests. ART is also provided at the health facility, and again the female dominated space may discourage men from accessing treatment.

A study in South Africa found that male dominated spaces such as health facilities servicing truck stop drivers and men working in the mining industry were more successful in engaging men [36]. One study in South Africa found that mobile, population wide testing via community health campaigns and home-based HIV testing reduced but did not eliminate barriers to male engagement in mobile community HIV testing [37]. New research has shown that testing at home with self-test kits is well accepted by men, however, this is a fairly new practice [37].

While these two theories offer different perspectives, they are also highly interconnected, as utilizing a ‘female space’ may threaten a man’s masculinity and deter him from utilizing the health facility. Both theories contribute to understanding men’s challenges engaging in HIV testing and treatment; however, they are insufficient to fully explain men’s aversion to HIV and ART.

### Echoes of the old art paradigm

Throughout this study and the literature, men’s fear of HIV is well documented. Kurt Riezler argues that fear may be shaped by ignorance [41] and this is particularly relevant in the context of HIV. Many men exhibit a lack of knowledge regarding HIV and ART [38]. This warrants a deeper examination, are men’s apprehensions towards HIV testing and treatment rooted in lack of information and education about the new developments in HIV care? While there has been tremendous progress in the ability for one to live a long and healthy life on ART and significant developments in simplification of ART, data from this study demonstrates lack of information about these developments among men and the community at large. This raises the question of whether men are rejecting HIV testing and treatment based on old associations of HIV and ART and what are effective means of communicating and engaging with men.

Results from this study show that many of the earlier truths about HIV and ART continue to persist. Old ideas of HIV as a death sentence continue to linger and beliefs such as these continue to discourage men from successfully engaging in HIV testing and treatment [11, 38]. Fear of visible side effects from the earlier drug regimens (primarily stavudine) were documented in this study and have been seen in other literature [42]. Study participants indicated that men have only learned about the simplified drug regimen from others on ART and that this information has not been shared widely in the community. Beliefs that ART is only for ‘very sick people’ continue to linger despite recent guidelines recommending that all HIV-positive people initiate ART irrespective of a CD4 count or clinical staging. Such beliefs may prevent men from engaging in HIV testing and treatment. Previous research has documented husband's interfere with their partner's adherence to ART [32]. In response to men’s attitudes towards HIV and ART, some women may choose to keep their HIV status a secret. However, failure to disclose one’s HIV status has been associated with poor adherence to ART [43].

In both countries, study participants spoke frequently about the need to further educate men about HIV and ART. Men lack information about living positively with HIV and the benefits of ART. Strengthening men’s acceptance of HIV testing and treatment will not only strengthen the men’s health, but it will also address women’s HIV vulnerability and enhance overall health outcomes in women [38].

Many efforts to increase uptake of HIV testing and treatment have focused on populations as a whole without paying attention to the specific needs and concerns of different populations within communities. Literature has documented that interventions are most successful when tailored and targeted to specific groups within the community [9]. Messages about HIV testing and ART need to target men and address their specific needs and concerns in places where men feel comfortable [19]. Previous research has indicated men’s interest in receiving information in a male dominated environment, from male HCWs, in the privacy of their homes and through information directed at them (not via their partners) [17, 18, 38].

In addition to providing accurate information about HIV and ART, there is a need to address gender norms, which discourages men from seeking health services and supporting their partners. Masculinity norms can be interpreted as a positive resource and be built upon in an HIV prevention context. Gender transformation programs such as the South African One Man Can or Stepping Stones [40, 44] aim to shift masculinity norms to encourage men to support their partner’s health as well as be an active agent in their own health care. Because gender norms are created and reinforced within the community by both men and women, these norms must be addressed not only at the individual level, but also at the community level. A study in Uganda highlighted how norms of masculinity can encourage receipt of HIV care and treatment, since it enabled HIV-positive men to feel strong, healthy and continue to work and provide for their family [45]. These gender transformation programs highlight agency and the ability for individuals and groups to redefine their masculinity. As Connell has contended there are different masculinities [34]. Different groups will have different interpretations of what masculinity is. It’s critical therefore to examine and address masculinity norms which undermine HIV testing and treatment within their social and cultural context.

### Limitations

The main strength of this study is the triangulation of the findings between different country contexts and different types of study participants. Similar findings from different country contexts increases the transferability of study results. Triangulating data from different types of study participants and comparing responses increases the validity of the results. The study had several limitations. The primary limitation is that no data were collected directly with men, all reports of men’s opinions and behaviors were reported from female study participants and HCWs. There are limitations to understanding one group’s beliefs and attitudes through another group’s interpretation. However, women's perspectives may provide more candor into men's fear of HIV and critical insight into how men influence women’s ART initiation and adherence. Another major limitation was the fact that women who participated in the study were recruited directly from the health facility and had initiated ART. Thus, the population does not include more vulnerable populations of women who may not have access to a health facility, who rejected Option B+, whose child did not live or who were unable to breastfeed their child. A final limitation was the simultaneous transcription and translation of the data, translating the data first and then transcribing reduces chances of interpretation errors.

## Conclusion

While universal ART has many benefits for individual health and aims to decrease the general HIV epidemic with treatment as prevention, there are many challenges that need to be overcome first. This study found that one of the primary barriers to women initiating and adhering to ART were their male partners. Fear of loss of masculinity and challenges accessing a female-centric facility are key factors that contribute to men’s aversion to engaging in HIV care and treatment. Outdated beliefs about HIV and ART also discourage men from utilizing HIV testing and treatment services. More information about HIV and ART is needed at the community level, and targeted towards the different community members, specifically men. Providing HIV testing and treatment services in male friendly spaces or at home may encourage men to accept HIV testing and treatment. Universal ART offers a unique opportunity to curb the epidemic, but successful implementation of these new guidelines is dependent on ART initiation and adherence by both women and men.

## Abbreviations

AIDS: Acquired immunodeficiency syndrome
ANC: Antenatal care
ART: Antiretroviral therapy
ARV: Antiretroviral drug
CD4: Cluster of differentiation 4
FGD: Focus group discussion
HCW: Health care worker
HIV: Human immunodeficiency virus
IDI: In-depth interview
MTCT: Mother-to-child transmission
PMTCT: Prevention of mother-to-child transmission
RA: Research assistant
TasP: Treatment as prevention
WHO: World Health Organization
